# Virulence assessment of *Listeria monocytogenes* grown in different foods using a *Galleria mellonella* model

**DOI:** 10.1371/journal.pone.0232485

**Published:** 2020-05-01

**Authors:** Mira Rakic Martinez, Martine Ferguson, Atin R. Datta

**Affiliations:** Center for Food Safety and Applied Nutrition, Food and Drug Administration, Laurel, MD, United States of America; Maria Curie-Sklodowska University, POLAND

## Abstract

Various produce including cantaloupe, caramel-coated apples, and packaged salads, have been recognized in recent years as vehicles for listeriosis, a human foodborne disease caused by intracellular pathogen *Listeria monocytogenes*. Our knowledge regarding the role of these foods in *L*. *monocytogenes* virulence, however, is limited. Understanding their role in modulating *L*. *monocytogenes* virulence can be useful in risk assessments and for developing control measures. In this study, we employed the *Galleria mellonella* larvae model to evaluate virulence potential of fifteen clinical, environmental and food isolates of *L*. *monocytogenes*, related to three major outbreaks, after growth on different foods. The non-human pathogen *Listeria innocua* was also included in the panel. Strains were inoculated in parallel in 5ml of brain heart infusion (BHI) broth, and on the surfaces of cantaloupe and apple fragments (5g each) at about 10^5^ colony forming units (CFU)/ml/fragment. One set of inoculated broth and food fragments was incubated at 10°C for 5 days while the second set was kept at 25°C for 3 days. *L*. *monocytogenes* cells were recovered from the fruits and BHI, washed twice, re-suspended in saline, and used to inoculate *G*. *mellonella* larvae at final concentrations of 10^6^ and 10^5^ CFU/larva. The larvae were incubated at 37°C and monitored for mortality (LT_50_—time taken to kill 50% of the larvae) and phenotypic changes over seven days. *L*. *monocytogenes* grown on cantaloupe and apple flesh surfaces resulted in higher virulence than when grown in BHI. *L*. *monocytogenes* infection at 10^6^ CFU/larvae resulted in an average LT_50_ of ≤ 30, 36 and 47 hours on cantaloupe, apples and BHI, respectively. These results represent a 2.5–4-fold increased mortality compared with an LT_50_ ≥120 hours in larvae infected with the same doses of *L*. *innocua* grown in corresponding matrices. Similar trends were also recorded with doses of about 10^5^ CFU /larvae.

## Introduction

Foodborne listeriosis, caused by the facultative intracellular pathogen *L*. *monocytogenes*, accounts for about 1600 illnesses and 250 deaths per year in the US [1]. Although the total number of illnesses are relatively small compared to that of other microbial foodborne diseases [1], the severity of the symptoms (e.g., septicemia, meningitis, stillbirths), high fatality rate (20–30%) and very high hospitalization rate (>95%) [2] that are associated with disease present significant challenges to public health, food safety regulators and industry. Although *L*. *monocytogenes* strains can be grouped into 13 serotypes, serotypes 1/2a, 1/2b and 4b [3] are known to account for the majority of human listeriosis cases and usually are related to soft cheeses and deli meats. However, an increasing number of listeriosis outbreaks related to previously unreported food vehicles [3–10] have been described in recent years. In 2010, the Texas Department of Health reported an outbreak of listeriosis in a health care facility that resulted in 10 infected patients and 5 deaths [5]. The cause of the outbreak was traced back to chicken salad and diced celery which was one of the ingredients of the salad. The unusually high number (50%) of deaths associated with this outbreak could be attributed to the immunocompromised status of patients since the outbreak was hospital-acquired and/or high number of *L*. *monocytogenes* in the chicken salad [6] which was shown to support *Listeria* growth [11].

Cantaloupes have been the causative agent of more than 19 outbreaks of foodborne listeriosis in the USA [12]; however, up until a major outbreak that occurred in 2011, the number of cases related to this fruit was not fully recognized. The reasons may include the long incubation period for the disease and a relatively short shelf life of the produce. The fatality rate of the 2011 outbreak related to cantaloupes, coupled with the development of invasive listeriosis (meningitis, septicemia) among otherwise healthy children, age 5–15 years, that occurred during a multistate outbreak related to pre-packed caramel apples [5] raised questions about the role of different foods on *L monocytogenes* virulence.

Due to the improved epidemiological surveillance and next generation whole genome sequencing number of outbreaks associated with previously unreported foods has significantly increased and may signal a potential of *L monocytogenes* to adapt and survive in a wide variety of foods previously not associated with listeriosis outbreaks. Our knowledge on mechanisms involved in such adaptation and the downstream effect on an organism’s pathogenicity and virulence potential following survival, adaptation and growth, are limited. Usually, infections caused by *L*. *monocytogenes* are asymptomatic or self-limited in healthy populations [13]. Ability of pathogens to survive under the stressful conditions of host gastrointestinal tract and possibility of adaptation to the said conditions may be contributing factor to the increasing number of illnesses in healthy population [14]. On the other hand, susceptible populations including the elderly, pregnant women or immunosuppressed individuals, could develop an invasive form of listeriosis with severe complications and death. Global transcriptome analysis of *L*. *monocytogenes* conducted by Kang et al. [15] has established that different food matrices affect its survival, growth and physiology. Some of these adaptive changes could be due to intrinsic food components and others may be extrinsic in nature e.g. preservatives added to food and storage temperature. It is however not clear how different food matrices affect the pathogenicity and virulence of *L*. *monocytogenes*.

The infection model of the greater wax moth *G*. *mellonella* was employed in various studies, as an alternative animal model, to monitor pathogenesis and virulence of human pathogens including *L*. *monocytogenes* [16–18]. With the advantages of cost efficacy, ease of manipulation, convenient growth conditions, ethical acceptability, and most importantly, the ability to incubate larvae at 37°C, an optimal temperature for the expression of major virulence factors of several bacterial pathogens including *L*. *monocytogenes*, *G*. *mellonella* larvae present a useful model for the assessment of its virulence and pathogenicity.

Our objective in this study was to analyze virulence of 15 *L*. *monocytogenes* strains belonging to three major disease-causing serotypes, 1/2a, 1/2b and 4b, following growth on cantaloupe and apple slices, and in BHI broth using the *G*. *mellonella* infection model. We also studied the effect of these food matrices at low temperature (10°C) growth as many produce commodities are routinely stored at low temperature as well as at room temperature. An understanding of the effects of these matrices on *L*. *monocytogenes* virulence would be immensely valuable in listeriosis risk assessment and food risk pairing and may assist in formulating policy guidance.

## Materials and methods

### *Listeria* spp. strains and inoculum preparation

Fifteen clinical, food and environmental isolates of *L*. *monocytogenes* related to three major outbreaks of listeriosis in the USA were evaluated in this study. The non-human pathogen *Listeria innocua* (LS9) was included in the panel as well (Table 1). *L*. *monocytogenes* strains belong to serotype 1/2a, 1/2b, 4b as well as 4bV [19], a variant of serotype 4b associated with three recent listeriosis outbreaks including the outbreak related to caramel-apples. Cultures were maintained at -80°C in brain and heart infusion (BHI) broth (Becton, Dickinson and Company, Sparks, MD, US) supplemented with 20% (v/v) glycerol. The frozen cultures were streaked onto Rapid L’Mono agar (BioRad, Hercules, CA) and incubated at 37°C for 24 h; a single colony was inoculated into BHI broth at 37°C for 24 h with shaking (175 rpm). The cells were collected by centrifugation at 4°C, rinsed twice and suspended in 5 ml of 0.85% sterile saline solution.

**Table 1:** *Listeria* strains employed in this study.

Strain No	Serotype	*Listeria* spp.	Source	Associated Outbreak
LS9	NA	*Listeria* spp.	Italian soft cheese	NA
LS411	4b	*L*. *monocytogenes*	Cheese	Jalisco cheese, California 1985
LS412	4b	*L*. *monocytogenes*	Clinical	Jalisco cheese, California 1985
LS670	1/2b	*L*. *monocytogenes*	Cantaloupe	Cantaloupe, USA 2011
LS672	1/2b	*L*. *monocytogenes*	Cantaloupe	Cantaloupe, USA 2011
LS675	1/2b	*L*. *monocytogenes*	Environmental	Cantaloupe, USA 2011
LS692	1/2a	*L*. *monocytogenes*	Environmental	Cantaloupe, USA 2011
LS740	1/2b	*L*. *monocytogenes*	Clinical	Cantaloupe, USA 2011
LS745	1/2a	*L*. *monocytogenes*	Clinical	Cantaloupe, USA 2011
LS1018	4b	*L*. *monocytogenes*	Clinical	Caramel apples, USA 2014
LS1026	4b	*L*. *monocytogenes*	Clinical	Caramel apples, USA 2014
LS1047	4bV	*L*. *monocytogenes*	Granny Smith apple	Caramel apples, USA 2014
LS1054	4b	*L*. *monocytogenes*	Granny Smith apple	Caramel apples, USA 2014
LS1055	4bV	*L*. *monocytogenes*	Granny Smith apple	Caramel apples, USA 2014
LS1058	4b	*L*. *monocytogenes*	Granny Smith apple	Caramel apples, USA 2014

NA—not applicable

### Growth of *Listeria* in BHI broth and on apple and cantaloupe fragments

Conventional (not organic) cantaloupes and apples (Fuji variety) were purchased at a grocery store. The pH of BHI broth and of fruit surfaces was measured by using two different types of probes (Routine Pro-ISM & Solid Pro-ISM). Using a sterile knife, the cantaloupe and apples were cut into slices, the rind removed, and the mesocarp cut into fragments (approx. 5g/fragment). Fragments were inoculated with individual *L*.*innocua* and *L*. *monocytogenes* strains at a concentration of about 10^5^CFU/ml/fragment. After incubation for either five days at 10°C or three days at 25°C, *L*.*innocua* and *L*. *monocytogenes* cells were recovered from the fruit by rinsing in 5 ml of 0.85% NaCl (saline) for 1 min to homogenize the samples. The saline rinses and BHI broth culture were centrifuged (high speed, 4°C) and the pellet was washed twice using the same volume of sterile saline solution. The final cell concentrations of these samples, as measured by CFU, were between 8.0 and 9.0 log CFU/ml/g. The *L*.*innocua* and *L*. *monocytogenes* populations in BHI, apple and cantaloupe samples were determined by plate counts on BHI agar and RAPID’ L. mono (RLM; Bio-Rad, Hercules, CA). Both BHI and RLM plates were incubated at 37°C for 24 h prior to counting colonies.

### Inoculation of *G*. *mellonella* larvae

Appropriate dilutions in saline were used to inoculate *Galleria* larvae (last instar *Galleria mellonella* larvae purchased from Vanderhorst Wholesale, Inc, Saint Mary, OH) with *L*.*innocua* and *L*. *monocytogenes*, at final concentrations of 10^6^ and 10^5^CFU/larva. A set of 20 *Galleria* larvae, obtained from the same batch, were inoculated in the last left pro-leg with 10μl of each dilution. Inoculated larvae were incubated at 37°C and monitored for mortality and phenotypic changes (changes of color, motility, dryness or pupation) over seven days. For each treatment, the LT_50_ (time taken to kill 50% of the larvae) and overall survival time (number of dead larvae at the end of the seven-day incubation at 37°C) were recorded. The LT_50_ indicates virulence of the pathogen, while the median overall survival refers to the length of time the larvae survived after inoculation with the pathogen. For each trial, one set of 10 un-inoculated larvae and one set of 10 larvae inoculated with sterile 0.85% saline solution were included. Group of un-inoculated larvae served as a control of adaptation of *Galleria* larvae to the temperature of 37°C, while second group served as a “manipulation” control. Experiment was conducted in two independent trials.

### Changes in populations of *Listeria* isolates in inoculated *G*. *mellonella* larvae

Changes were assessed as previously described [18]. Briefly, at the time points of 2- and 24-hours post-inoculation, five surviving larvae from each tested group were selected, surface-sterilized and crushed in 1ml of sterile saline. Serial dilutions of these samples were plated on to RAPID’*L*.*mono* Medium (BIO RAD, USA) agar and incubated at 37°C for 24–36 h before enumeration of typical *L*. *monocytogenes* colonies.

#### Statistical analysis

Kaplan Meier was used to estimate the survival functions and the Wilcoxon chi-square test was used to test for equality of survival functions.

## Results

### Growth of *L*. *monocytogenes* and *L*. *innocua* in BHI, apple and cantaloupe fragments

**Fig 1:**
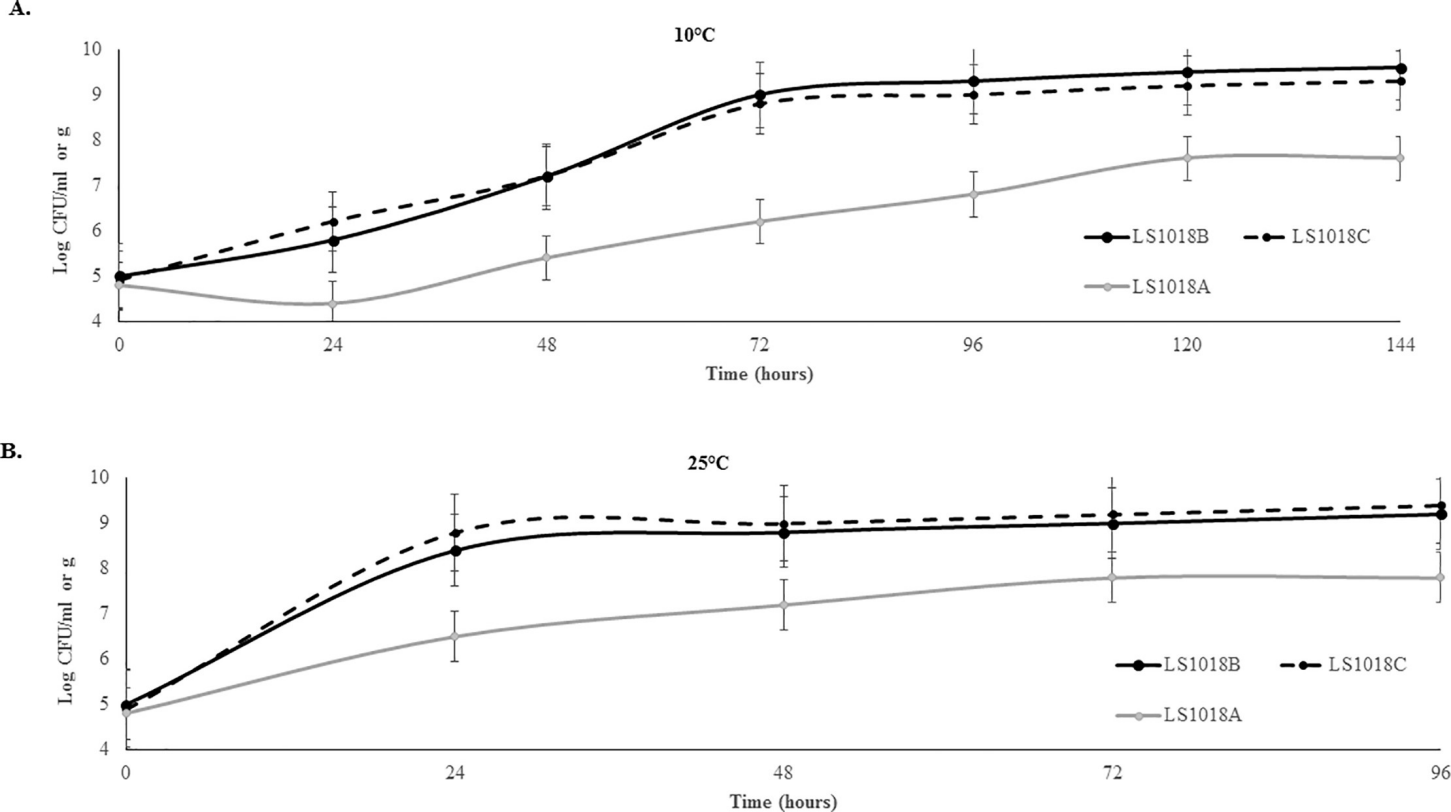
Growth of *L*. *monocytogenes* LS1018 in BHI, on cantaloupe or on apple fragments at 10°C and 25°C. Results are from two independent experiments; error bars represent a mean ±SD.

Growth curves of *L*. *monocytogenes* strains grown in BHI, on cantaloupe, and on apple fragments were generated at 10°C and 25°C. The temperatures were chosen to reflect produce storage temperatures at retail and home settings. Results show comparable growth rates in BHI and cantaloupe at both tested temperatures. After 5 days at 10°C *L*. *monocytogenes* reached stationary phase on both produce and in BHI with population densities of 9.5 log CFU/ml 9.2 log CFU/g in BHI and cantaloupes, respectively (Fig 1A). The *L*. *monocytogenes* strain on apples grew slower with an initial decline within the first 24 hours at 10°C followed by recovery and growth to approx. 7.6 log CFU/g by the end of incubation period (Fig 1A). At 25°C, *L*. *monocytogenes* reached stationary phase after 72 hours of incubation (Fig 1B) with a population density of approx. 9.2 CFU/ml of g in BHI and cantaloupe slices of 7.8 CFU/g in apple slices. Growth of *L*. *innocua* (LS9) in BHI, on apples and cantaloupe was comparable to the growth of *L*. *monocytogenes* at each tested temperature (data not shown). Recorded pH values for BHI, cantaloupe, and apple chunks prior to the inoculation were 7.4, 6.5 and 3.8, respectively.

### Monitoring of *Galleria mellonella* larvae

**Fig 2:**
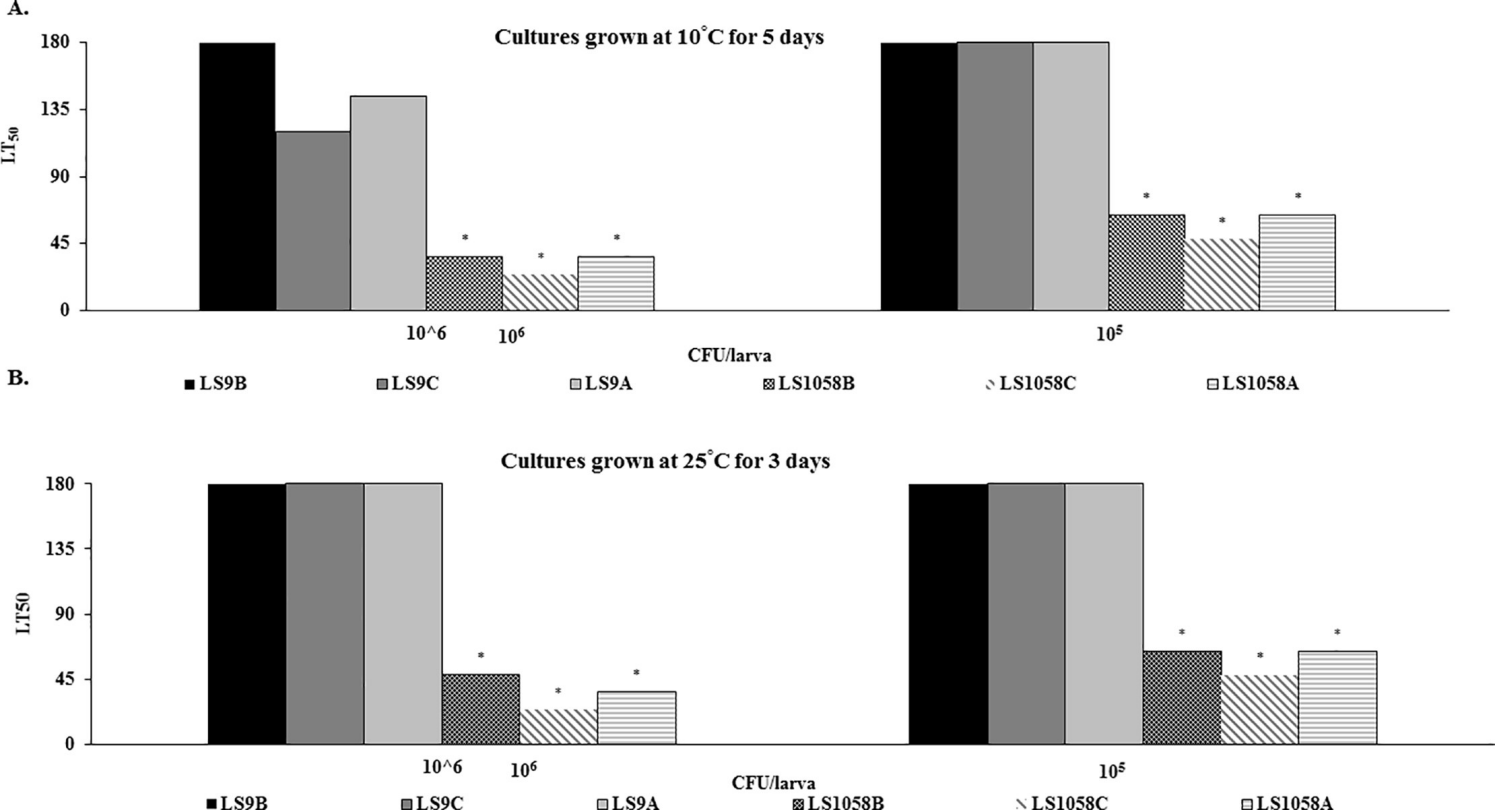
Lethal time necessary to kill half of larvae (LT50) inoculated with *L*. *monocytogenes* cells grown in either BHI (●), cantaloupe (■) or apple fragments (Δ). An * indicates a significantly *(p < .0001) longer time needed to kill half of larvae inoculated with non-human pathogen *L*. *innocua* recovered from BHI (●), cantaloupe (■) and apple (Δ) incubated at (A) 10°C and (B) 25°C, respectively. An * * indicates that we were not able to establish LT_50_ values for *L*. *innocua* since number of dead larvae did not reach 50% over the monitoring period of seven days.

*Galleria* larvae inoculated with *L*. *monocytogenes* had significantly (p < .05) higher mortality than larvae inoculated with non-pathogenic *L*. *innocua*, over the period of seven days, regardless of the dose, growth temperature, or growth matrix. At the doses of 10^6^CFU/larva we were not able to establish LT_50_ values for the *L*. *innocua* grown in BHI prior to the infection, since majority of larvae were alive at the end of the experiment (Fig 2A). At the doses of 10^5^CFU/larva, *L*. *innocua* recovered from any of the tested matrices did not kill 50% of larvae by the end of the experiment (Fig 2B). The two doses used in this study had different survival outcomes with the higher dose (10^6^CFU/larva) resulting in a shorter LT50 values (higher virulence) then the lower dose (10^5^CFU/larva). Comparing strains, while stratifying by source and dose, results revealed significantly different (p < .05) survival outcomes that were strain-related. Among the strains tested simultaneously on cantaloupe and in BHI, but not on apple fragments, the strain LS740 had the shortest mean survival time (i.e. most virulent) at dose 10^6^ CFU/larva while the strain LS670 had the shortest mean survival time at 10^5^CFU/larva. Strain LS692 had the highest mean survival times (i.e. least virulent) at both doses. Strain related differences were also observed among the 6 *L*. *monocytogenes* strains simultaneously tested on all matrices. Non-pathogenic *L*. *innocua* LS9 had the highest mean survival times (i.e. least virulent) regardless of doses or source when compared with *L*. *monocytogenes*.

**Fig 3:**
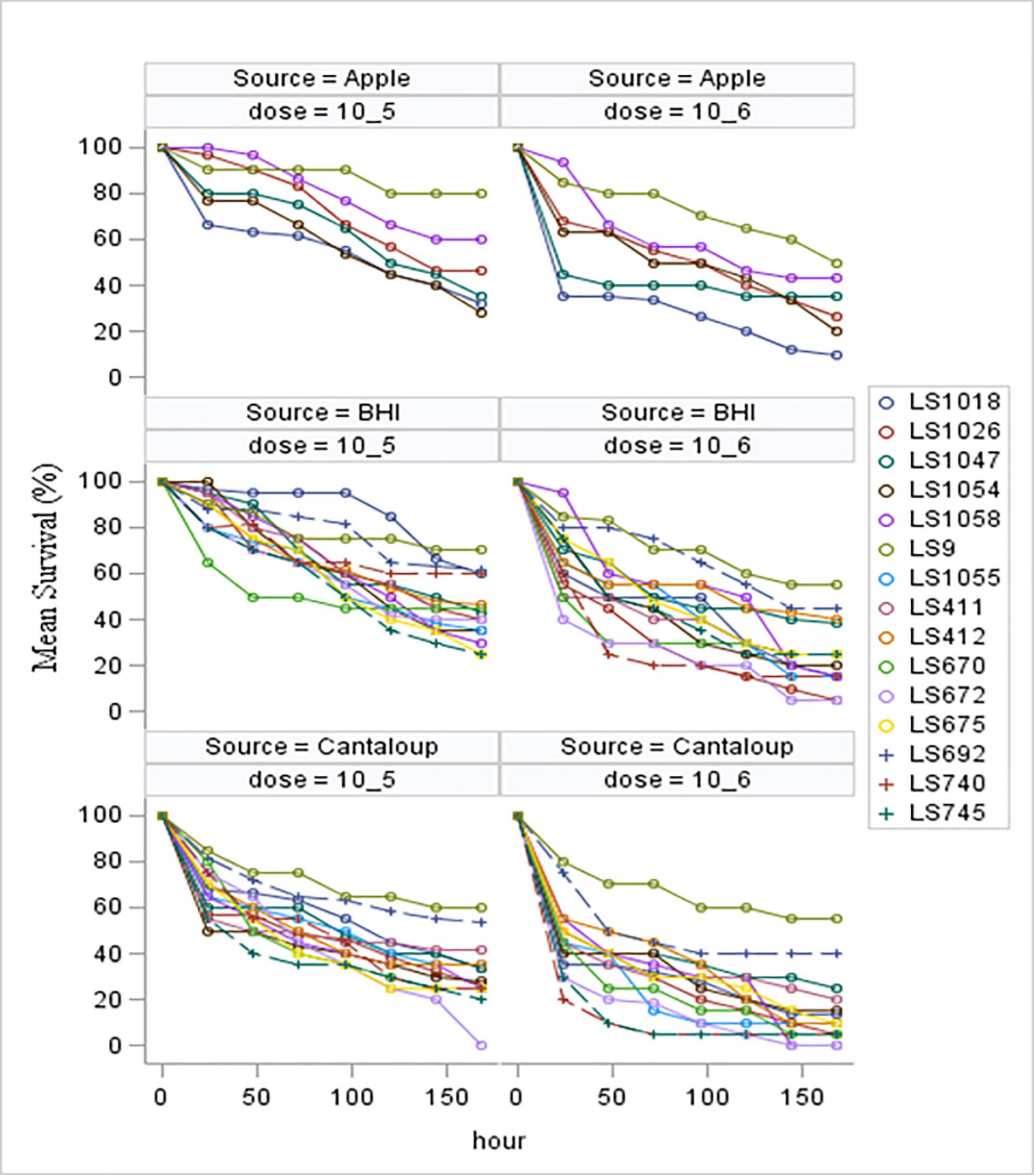
Virulence of *L*. *monocytogenes* strains (represented as LT_50_) in *Galleria* larvae inoculated with the pathogen at the concentrations of (A) 10^6^CFU/larva and (B) 10^5^CFU/larva. Prior to the inoculation, Lm was recovered from BHI (●), cantaloupe fragments (■) and apple fragments (Δ), separately, after 5 days at 10° C. Nonpathogenic *L*. *innocua* was also included in the panel, but we were not able to establish LT_50_ values since number of dead larvae did not reach 50% over the monitoring period of seven days.

Comparing growth matrices, while stratifying by dose (10^5^ or 10^6^CFU/larva) and strain, *L*. *monocytogenes* cells recovered from cantaloupe, on average had significantly (p < .0001) shorter LT_50_ values (higher virulence). Virulence of *L*. *monocytogenes* cells recovered from apples was also strain and dose related. One strain recovered from apple had a significantly (p < .0001) longer LT_50_ value than the same strain recovered from cantaloupe at the dose of 10^6^CFU/larva, while five strains had significantly (p < .0001) longer LT_50_ values_,_ at a dose of 10^5^CFU/larva. On the other hand, three strains recovered from apples had significantly lower (p < .0001) LT_50_ values when compared with those recovered from BHI at the doses of 10^6^CFU/larva and one strain (LS1058) at a dose of 10^5^CFU/larva. Individual LT_50_ values for strains grown on cantaloupe, apples and in BHI are shown in Table 2. Virulence of *L*. *monocytogenes* strains initially grown on cantaloupe, apples, and in BHI and subsequently used to inoculate *Galleria* larvae reflected through LT_50_ values are shown in Fig 3. Infection with higher dose of 10^6^ CFU/larva resulted with a significantly (p < .0001) lower LT_50_ value and mean overall survival time. Cells recovered from BHI broth (B) had significantly (p < .0001) higher LT_50_ values and mean survival time than those recovered from cantaloupe (C); One of the *L*. *monocytogenes* strains recovered from apples (A) had significantly (p < .0001) higher LT_50_ value when compared with the same strain recovered from cantaloupe, while three of the strains recovered from apples had significantly (p < .0001) lower LT_50_ value than those recovered from BHI.

**Table 2:** LT_50_ values of different *L*. *monocytogenes* and *L*. *innocua* (LS9) strains grown on cantaloupe fragments (C), apple fragments (A) and BHI at 10°C and 25°C.

LT_50_ (h)* values												
Strain No.		Grown at 10°C for 5 days					Grown at 25°C for 3 days					
	C	A	BHI	C	A	BHI	C	A	BHI	C	A	BHI
	10^6^ CFU/larva			10^5^ CFU/larva			10^6^ CFU/larva			10^5^ CFU/larva		
LS9	120±10	144±2	≥168	≥168	≥168	≥168	≥168	≥168	≥168	≥168	≥168	≥168
LS411	24±0	NT	48±0	120±4	NT	144±2	40±4	NT	80±2	96±0	NT	130±2
LS412	18±4	NT	24±2	72±0	NT	80±4	18±4	NT	48±0	80±4	NT	120±0
LS670	18±4	NT	48±4	48±0	NT	72±0	18±2	NT	60±4	130±2	NT	≥168
LS672	18±4	NT	24±4	48±4	NT	72±0	24±4	NT	30±4	96±0	NT	120±0
LS675	18±4	NT	30±0	72±2	NT	96±0	18±2	NT	40±2	72±0	NT	120±0
LS692	48±0	NT	72±0	120±0	NT	144±2	80±4	NT	96±0	96±0	NT	120±0
LS740	30±0	30±4	36±4	48±0	60±4	72±0	24±0	36±4	40±4	48±0	48±0	72±0
LS745	18±2	NT	24±0	48±0	NT	60±4	18±4	NT	24±0	60±4	NT	72±0
LS1018	48±2	54±2	72±0	64±6	72±0	96±0	48±0	60±2	72±0	72±0	80±4	96±0
LS1026	24±2	24±0	30±2	48±2	64±2	72±0	30±4	40±4	48±0	64±0	72±0	80±4
LS1047	30±2	48±0	72±0	56±4	80±2	120±4	36±4	48±0	72±0	64±0	96±0	120±0
LS1054	24±	24±0	72±0	48±0	64±4	96±2	36±3	48±0	80±4	64±0	72±0	120±0
LS1055	36±2	36±4	48±4	56±4	72±0	96±0	48±0	48±0	60±4	72±0	80±4	96±0
LS1058	24±0	36±2	48±0	60±4	64±2	64±4	36±2	48±0	56±2	60±4	64±2	80±4

*Experiments were performed in two independent trials and L_T50_ values presented as a mean ±SD. C: Cantaloupe fragment; A: Apple fragment; BHI: Brain heart infusion broth

**Fig 4:**
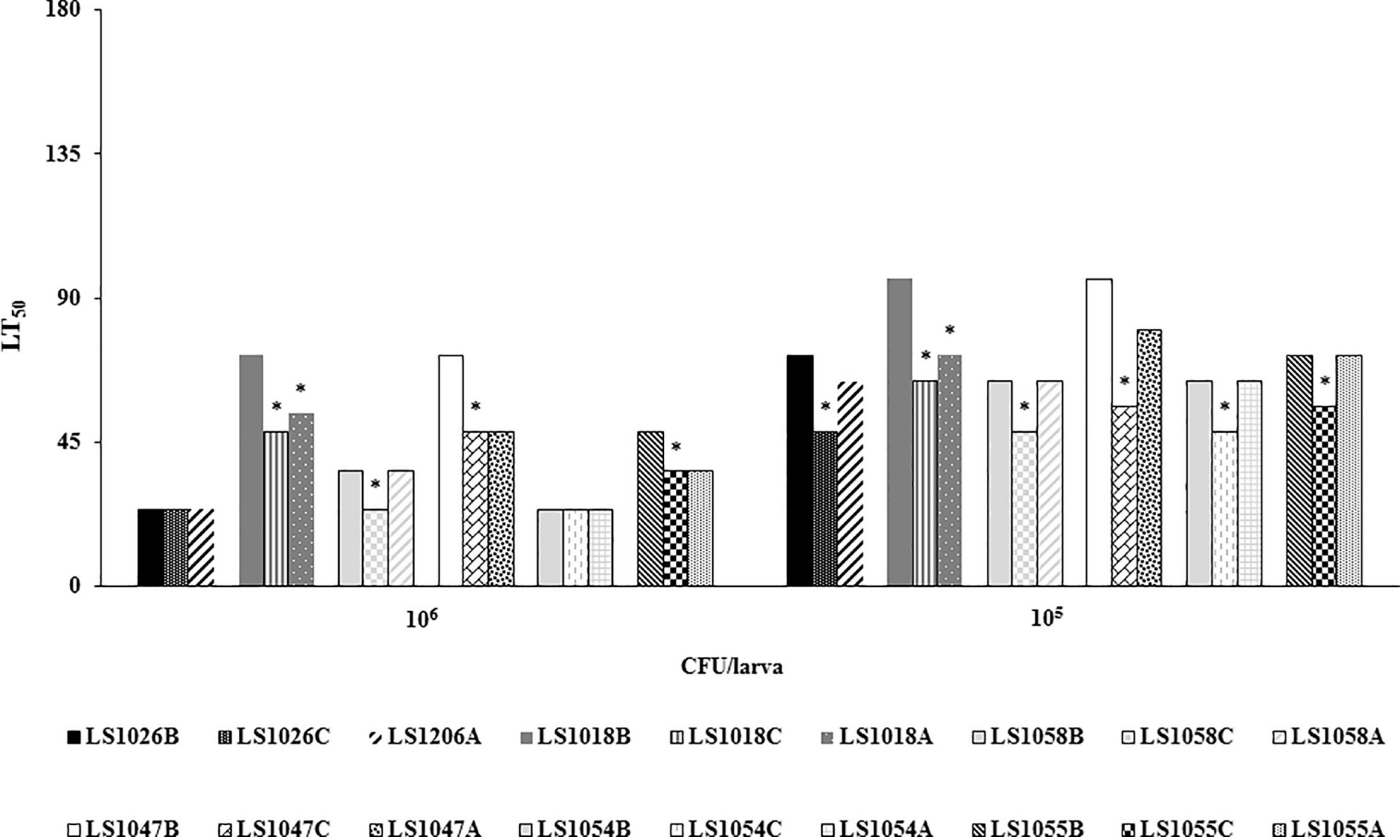
Mean survival of *Galleria mellonella* larvae following inoculation with *L*. *monocytogenes* grown in BHI, on apples and cantaloupe, averaged over all strain at the doses of (A) 10^6^CFU/larva and (B) 10^5^CFU/larva.

While some of the tested strains recovered from apples had shorter LT_50_ values than those recovered from BHI, the mean overall survival time of *Galleria* larvae inoculated with cells recovered from apples was longer. This difference was not statistically significant. Cells recovered from cantaloupe differed significantly (p < .0001), with the shortest mean overall survival time compared to cells recovered from BHI broth or from apple surfaces. The results were averaged across all strains and presented with the standard deviations in Table 3 and Fig 4.

**Table 3:** Mean survival time of *G*. *mellonella* larvae at 37°C: After inoculation with *L*. *monocytogenes* strains grown in BHI, on apple, or on cantaloupe fragments. The results were averaged across all strains. The table present the results with the standard deviations.

Source	Mean survival time (h), recorded over the 7 days post inoculation	
	10^6^ CFU/larva ± SD	10^5^ CFU/larva ±SD
Cantaloupe	114*±9	126*±6
Apple	127±12	143±10
BHI	124±9	140±8

* Larvae inoculated with *Listeria* cells recovered from cantaloupe had significantly (p < .0001) lower mean survival time when compared to apples or BHI.

### In-host change of *L*. *monocytogenes* population

To assess the extent of *Listeria* death and subsequent growth variability of different strains infecting *Galleria* larvae, we measured the total number of viable *Listeria* (CFU/larva) after 2h of incubation of the larvae post-inoculation. Two hours post inoculation the number of viable *Listeria* cells in *Galleria* at both inoculum concentrations (10^5^CFU/larva 10^6^CFU/larva) had decreased approx. 1–3 log units, depending on the strain and the matrix they were grown in prior to infection. The decrease was more notable in strains grown on cantaloupe and apples than in BHI, but these differences were not statistically significant. This was followed by cell recovery and growth to final counts of 6.0 to 9.0 log CFU/larva at 24h time point. None of the tested strains expressed significantly different changes in population densities compared to others. Our results also indicated that neither growth matrices nor growth temperature significantly influenced in-host change of pathogen population dynamics.

## Discussion

The emergence of previously un-reported foods as vehicles of recent listeriosis outbreaks [5, 6, 8, 10] and the change in demography of affected populations [5] revealed the necessity of better understanding of the effects of different foods on *L*. *monocytogenes* pathogenicity and virulence mechanisms. Such an understanding might help to improve risk assessments associated with this pathogen. However, available data regarding the role of food matrices in virulence of *L*. *monocytogenes* is limited [15, 20, 21]. In recent years, use of larvae of the greater wax moth *Galleria mellonella* has emerged as a promising model for the assessment of virulence of numerous human pathogens including *L*. *monocytogenes* [22]. Although *G*. *mellonella* model by-passes human gastrointestinal system, an important first line defense against foodborne pathogens, the innate immune system of *G*. *mellonella* resembles to one of mammal’s, with enzymes, reactive oxygen species and antimicrobial peptides necessary to protect from bacterial infection making it an attractive animal model for studying virulence of pathogenic bacteria [16]. Schrama et al., 2013 [23] evaluated the effect of acid and salt adaptation of *L monocytogenes* in a cheese-based medium on the virulence of pathogen using the *G*. *mellonella* model. Their results suggested that adaptation to different food-related factors can affect the virulence of *Listeria*. They observed overexpression of *inlA*, *actA* and *hly* genes in *L*. *monocytogenes* strains adapted to pH 5.5 and 3.5%w/v NaCl when compared to non-adapted cells. However, they concluded that expression of virulence genes in changed conditions (e.g. lower pH and change in salt content) was strain related [23]. Other findings also indicate that adaptation to acid and salts increased the ability of *L*. *monocytogenes* to invade Caco-2 cells and grow inside macrophages [24, 25, 26]. Results from several studies indicated that the changes in virulence gene expression, an indication of change in bacterial physiology, were due to different food matrices. Tang et al., 2015 [27] characterized the transcriptome of *L*. *monocytogenes* grown on cold smoked salmon (CSS) by comparing it with the transcriptome of the pathogen grown in modified BHI (MBHI) (4.65% NaCl and pH 6.1, levels normally encountered in CSS). Even though growth (rate as well as maximum cell counts) of *L*. *monocytogenes* was similar on CSS and in modified BHI, the results indicated higher transcript levels of PrfA controlled genes in cells grown on CCS than those grown in MBHI. These authors argued that restricted oxygen conditions could affect expression of the PrfA regulon. Limited oxygen conditions could also lead to higher invasion of Caco-cells as reported by Larsen et al., 2010 and others [28, 29, 30, 31]. Similarly, growth on cantaloupes has also been studied. Kang et al., 2019 [15] described up-regulation of *hly*, *plcA*, *plcB* and *actA* genes in an *L*. *monocytogenes* strain grown on cantaloupe when compared to the strain grown in BHI. These authors speculated that co-regulation of genes involved in metabolism and virulence of *L*. *monocytogenes* may be the cause for extensive changes in stress response and virulence genes expression. These results agree with a previous study reported by Hadjilouka et al., 2016 [32] who reported upregulation of *hly* and *plcA* genes in *L*. *monocytogenes* isolates recovered from melons in comparison with those recovered from BHI, while *plcB* expression varied depending on temperature. This group was studying key *L*. *monocytogenes* virulence genes during growth on melon and BHI at different temperatures [32].

Increased demand for and consumption of fresh produce in the USA in recent years led to the significant increase of foodborne outbreaks related to these foods [33]. Over 34 foodborne outbreaks involving melons have been recorded during the last two decades [12], with the most lethal multistate outbreak of listeriosis related to contaminated cantaloupes that occurred in 2011. This multistate cantaloupe-associated outbreak resulted in 33 deaths, 147 illnesses and 1 miscarriage [7]. With low acidity (high pH), high water activity [12] and sugar content, cantaloupes present an ideal medium for growth of microorganisms including *L*. *monocytogenes*. In fact, several studies have shown that strains of *L*. *monocytogenes* grew very well in cantaloupe flesh at 4°C, 10°C as well as at 25°C and the growth rate and maximum growth were on par with BHI [15, 34]. We evaluated the potential change in virulence of 14 *L*. *monocytogenes* strains and one *L*. *innocua* strain grown at different temperatures on cantaloupe fragments. The results show that the cells recovered from cantaloupe had significantly (p < .0001) lower LT_50_ values and mean survival time than cells recovered from BHI broth which indicates higher virulence of these cells. A possible explanation could be the increased transcription of stress and virulence related genes of *L*. *monocytogenes* after growth on cantaloupe as reported by Kang et al., 2019 [15] who also reported increased transcription of the core PrfA regulon except for the *prfA* gene. The PrfA transcriptional activator regulates genes encoding major virulence factors in *L*. *monocytogenes*, including phospholipases PlcA and PlcB, and the pore-forming toxin Hly [35]. PrfA regulated genes are strongly induced during infection [35, 36, 37]. Available studies [16, 18, 38] have found that deletion of these genes in *L*. *monocytogenes*, significantly decreased mortality of *Galleria mellonella* larvae, which is why the upregulation of these genes after growth on cantaloupe could result in higher strain virulence. The extent of the difference between the cantaloupe grown and BHI grown cells were found to be strain and dose dependent. Although the total number of cells inside *Galleria* after 24h post-inoculation was lower for cantaloupe grown *L*. *monocytogenes* compared to BHI, the consistent pattern of increased virulence among all strains indicate that the observed difference in virulence was not due to increase in number of cells but rather a change in virulence gene expression. In-fact, if we consider infection outcomes (LT_50_ values and mean survival) per-cell basis, the effect on cantaloupe grown cells appear to be much higher than the cells grown in BHI. Our findings are also supported by the findings by Kang et al., 2019 [15], who reported that growth of *L*. *monocytogenes* in cantaloupe resulted with altered pathogen virulence gene expression. Furthermore, this group also showed enhanced survival of *L*. *monocytogenes* strains grown on cantaloupe in the synthetic gastric fluid model, which indicate that cantaloupe-grown *L*. *monocytogenes* cells have better chance of survival during passage through the stomach gastric barrier and development of infection [15].

Apples were not previously considered as vehicle for listeriosis until the outbreak that occurred in 2014 [5]. This outbreak was linked to the consumption of caramel-coated apples which sickened 35 people across the USA and in Canada and included three deaths. Even though most of the cases during this outbreak occurred in the Midwest and southwestern USA, *L*. *monocytogenes* isolates related to this outbreak, analyzed by whole genome sequencing were indistinguishable from outbreak-associated isolate in Canada. This outbreak, among others, affected three otherwise healthy, young males, age 5–15, not a typical risk group for *L*. *monocytogenes*. It is interesting to note that a cohort study with microbiologically proven human listeriosis cases also identified about 4% neurolisteriosis cases, which had no comorbidity or ongoing pregnancy and were less than 40 years old [39]. One of the interesting findings related to strains of this outbreak was that one of the *L*. *monocytogenes* strains isolated from the contaminated apples was 4bV, a variant of serotype 4b (lineage I) with a 6.3kb DNA segment, typically linked to lineage II strains including serotype 1/2a, 1/2c, 3a and 3c [19]. Assessment of the virulence of *L*. *monocytogenes* in our study, which included both 4b and 4bV strains from the caramel apple outbreak, also included apple slices as a food matrix for *L*. *monocytogenes* growth. The pH value of inoculated apples throughout the experiment averaged at 3.9. Lower pH values (pH 5.5 and below) could trigger acquisition of different protective and cross protective mechanisms in *L*. *monocytogenes* [20, 22]. Such mechanisms could impact virulence as reported by Schrama et al. [23]. Their results showed lower mortality of *Galleria* larvae after inoculation with *L*. *monocytogenes* strains adapted to pH 5.5 and 3.5% NaCl in simulated cheese medium. In contrast, we have found that cells recovered from apples with similar pH values can cause significantly (p < .0001) higher mortality of *Galleria* larvae, but mean survival time was longer when compared to the cells recovered from BHI broth. The extent of these effects was strain related; strains LS1018, LS1047, and LS1055, recovered from apples, killed 50% of the larvae population in a shorter time than those recovered from BHI. However, surviving larvae lived longer than those inoculated with cells recovered from BHI. The contrast between these two studies could be due to either the different growth matrix or the absence of NaCl in our study. A lower incubation temperature (10°C) for apple slices compared to 22°C employed in the cheese study could also be of importance since acid-stress adaptation of *L*. *monocytogenes* does not occur at lower temperatures [40]. However, we have found similar trends in mortality of larvae after incubation of apple slices at 10°C and 25°C, hence our deduction that factors other than pH and temperature may be involved in the change of virulence of *L*. *monocytogenes* cells grown on apples. The virulence of *L*. *monocytogenes* strains grown on apples was, however, lower than of those strains grown on cantaloupe. Our data shows that in-host growth of *L*. *monocytogenes* was not affected by growth matrix nor storage temperature since there was no significant difference among pathogen populations after inoculation. This finding possibly reflects actual change in virulence rather than the in-host growth kinetics. The change in virulence could be prompted by a more beneficial environment on the cantaloupe flesh with neutral pH and higher sugar content. On the other hand, growth on apple fragments with lower pH values could cause a strong induction of virulence genes as a part of a defensive mechanism with limited time effect, which would lead to shorter LT_50_ values but longer mean overall survival when compared to the cells recovered from BHI as was recorded in the current study. Even though growth of *L*.*innocua* strain LS9, a non-pathogenic *Listeria control*, was comparable to the growth of *L*. *monocytogenes* strains on each of the tested matrix and condition, and also in *Galleria* larvae the strain did not affect *Galleria* survival.

Overall, in the *Galleria* insect larvae infection model, cantaloupe and apple grown *L*. *monocytogenes* resulted in higher virulence than *L*. *monocytogenes* grown in BHI demonstrating the significance of the food matrix in virulence. Further studies are necessary to determine the factors present in food that may affect the virulence of *L*. *monocytogenes* strains. The effects of other ready-to-eat foods also need to be evaluated and the results obtained from these studies need to be compared with real-life listeriosis outbreaks. Increased virulence may lead to a reduction in infective dose and/or increased severity of disease. Effects of different food matrices, either intrinsic or extrinsic, could also affect *L*. *monocytogenes* survival and growth and have a downstream effect on infection outcome. For example, it is well established that many foods contain relatively high levels of glycine-betaine and its analogues, known osmo and cryo protectant chemicals [41]. It is expected that these foods will provide higher levels of tolerance to *L*. *monocytogenes* when they are exposed to high salt and stored at lower temperature. Brown et al. have recently shown that nitrogen starvation can promote formation of ciprofloxacin-tolerant persisters [42]. If such a condition persists in any food matrix, the *L*. *monocytogenes cells* grown in these foods may be more refractory to standard antibiotic therapy leading to a more acute infection outcome. A thorough understanding of the effects of food matrices on growth, survival, virulence potential and infection outcome of *L*. *monocytogenes* may provide a better way to assess risk from foodborne listeriosis and may provide clues to formulate some preventative measures and control foodborne listeriosis.
